# Baduanjin exercise intervention for community adults at risk of ischamic stroke: A randomized controlled trial

**DOI:** 10.1038/s41598-018-37544-0

**Published:** 2019-02-04

**Authors:** Guohua Zheng, Bai Chen, Qianying Fang, Qiu Lin, Jing Tao, Lidian Chen

**Affiliations:** 1 College of Nursing and Health Management, Shanghai University of Medicine & Health Sciences, Shanghai, 201318 China; 2 College of Rehabilitation Medicine, Fujian University of Traditional Chinese Medicine, Fuzhou, 350122 China; 3 Department of Physical Education, Fujian University of Traditional Chinese Medicine, Fuzhou, 350122 China; 4 Fujian Key Laboratory of Rehabilitation Technology, Fujian University of Traditional Chinese Medicine, Fuzhou, 350122 China; 5 Collaborative Innovation Center for Rehabilitation Technology, Fujian University of Traditional Chinese Medicine, Fuzhou, 350122 China

## Abstract

The aim of current study was to assess the effects of Baduanjin exercise on cerebrovascular function, cardiac structure and cardiac function, static pulmonary function, traditional risk factors of CVD and the related psychological outcomes in older community adults at risk for ischaemic stroke. A randomized controlled trial was conducted in three community between November 2013 and October 2015. Older community-dwelling adults (N = 170) were randomly allocated into either a Baduanjin training (5 × 60 min/weekly) or control group who kept their unaltered lifestyle during a 12-week intervention period. Primary (cerebral haemodynamic parameters) and secondary outcomes (cardiac structure, cardiac function, static pulmonary function, traditional risk factors and the related psychological outcomes) were measured at baseline, after a 12-week intervention period and after an additional 12-week follow-up period. After the 12-week intervention period and additional 12-week follow-up period, the Baduanjin exercise group displayed significant changes in most cerebral haemodynamic parameters compared to the control group: lower systolic blood pressure, diastolic blood pressure, plasma total cholesterol levels, waist circumference, hip circumference and waist/hip ratio; and improved mood, self-confidence, self-esteem, quality of life and sleep quality. A supervised 12-week Baduanjin exercise intervention was effective and safe in modulating cerebral haemodynamics, reducing blood pressure and improving anthropometric parameters and related psychological outcomes in older community adults at risk for ischaemic stroke.

## Introduction

Ischaemic stroke remains the leading cause of long-term disability in adults worldwide^1^. Although age-standardized mortality of stroke have decreased around the world in the past twenty years, the absolute number of people suffering from stroke is increasing, and its impact is likely to dramatically increase in the future because of the ageing population and health transitions observed in developing countries^2,3^. Currently, no country in the world has exhibited a substantial reduction in stroke burden in terms of the absolute number of people affected by and/or dying from stroke. This implies that it is important to make efforts to prevent stroke, particularly the establishment of effective primary prevention strategies for older community adults.

Studies have demonstrated that stroke and most other cardiovascular diseases can be prevented^4^. The most effective means available for stroke prevention involve the control of modifiable risk factors^5,6^. According to the INTERSTROKE study, ten risk factors, including vascular risk factors, unhealthy diet and physical inactivity, account for 90% of the population-attributable risk of stroke, and most of them are modifiable risk factors^7,8^. It is well known that more than 50% of strokes could be prevented through the control of modifiable risk factors^9^. Even so, their preventive mechanisms remain unclear, which may limit the efficacy of primary prevention.

In terms of aetiology, the direct cause of ischaemic stroke is associated with intracranial atherosclerotic stenosis and the disruption of atherosclerotic plaques with subsequent thromboembolism^10^. The downstream cerebral haemodynamic changes and the cerebrovascular reserve may play an important role^11^. For example, the narrowing cerebral vasculature resulting from cerebral intracranial atherosclerosis can cause a reduction in cerebral perfusion pressure (CPP), and the subsequent autoregulation of the vasculature will maximally dilate the cerebral arterioles to maintain cerebral blood flow (CBF). However, with the further reduction in CPP and the maximally dilated arterioles, CBF will also decrease and potentially increase the risk of stroke^12^. Therefore, cerebral haemodynamic impairment is considered an important pathophysiological mechanism involved in ischaemic stroke. CBF depends on arterial blood pressure, cerebrovascular resistance, and the distribution of cardiac output. CBP is rigorously regulated by a set of mechanisms that include cerebral autoregulation, neurovascular coupling and cerebrovascular carbon dioxide and oxygen reactivity to safeguard the balance of cerebral metabolic demand and supply^13,14^. Studies have suggested that an increase in cerebrovascular resistance and a decrease in CPP could cause a reduction in cerebral blood flow^15^, whereas a slight alteration in cardiac output could also lead to changes in CBF despite blood pressure remaining stable or within the autoregulatory range^16^. Generally, traditional risk factors of ischaemic stroke, such as ageing, hypertension, diabetes, and lipid disorders, are associated with these conditions. For example, diabetes is associated with an increased risk of cerebral autoregulation dysfunction, whereas poorly controlled hypertension is related to a decline in CBF^17,18^. Normal ageing is linked to marked structural and functional alterations in the cerebrovascular system^19^. Recent studies have indicated that an estimated 30% of older community adults over 65 years old suffer from intracranial atherosclerotic stenosis (ICAS), as determined by magnetic resonance angiography^20^, and that more than 90% of individuals with ICAS have at least one vascular risk factor of cardiovascular disease (CVD)^21^. Therefore, controlling traditional cardiovascular risk factors and their appropriate treatment are vital to establishing primary preventive strategies of stroke by improving the direct aetiology of ischaemic stroke.

Current evidence clearly shows that exercise or physical activity is an effective intervention for preventing ischaemic stroke or other cardiovascular diseases. Therefore, the American Heart Association (AHA) and American College of Cardiology (ACC) recommend that adults should participate in at least 3 sessions of aerobic physical activity of moderate or vigorous intensity lasting an average of 40 minutes each week^22,23^. However, fewer than 50% of adults, particularly adults over the age of 60, achieve this recommendation despite the broad recognition of its benefits^24^. As one of the traditional Chinese Qigong exercises, Baduanjin exercise consists of eight separate, delicate and smooth movements; it is a low-moderate intensity mind-body exercise that has been practised in China for hundreds of years^25^. Current studies have suggested that Baduanjin training appears to have substantive benefits for older adults and is an appropriate exercise for older community people^26,27^. Many significant improvements have been reported in balance, strength and flexibility as well as in sleep quality and psychological and mental health^28–30^. Recently, an increasing number of studies have demonstrated the benefits of regular Baduanjin practise in reducing blood pressure, insulin resistance and blood glucose levels as well as in controlling traditional CVD risk factors^31–33^. However, the effects of Baduanjin exercise on the aetiology of ischaemic stroke, such as cerebral haemodynamics in older community people, have not been studied yet. The aim of this study was to conduct a randomized controlled trial to examine the effects of Baduanjin exercise on cerebrovascular functions, cardiac structure and function, static pulmonary function, physical fitness, traditional risk factors of CVD, quality of life and sleep quality in older community adults at risk for ischaemic stroke.

## Methods

### Design and Setting

This study was a two-armed, randomized, assessor-blinded, parallel controlled trial with computer-generated randomization and the concealed allocation. It was conducted between November 2013 and October 2015 in three community centres (Wufeng, Fengdanbailu, and Chuntian) in Fuzhou, China. The participants were randomly allocated to either a Baduanjin training group or usual physical activity group at a 1:1 ratio. Ethics approval was obtained from a local ethics committee in the Affiliated People’s Hospital of Fujian University of Traditional Chinese Medicine (No. 2013-021-02). This study was performed in accordance with the Declaration of Helsinki. The trial has been registered in the Chinese Clinical Trial Registry (ChiCTR-TRC-13003588). The design of this study was described in further detail in the published study protocol^34^.

### Participants

The participants were at a high risk of ischaemic stroke according to the “2012 annual screening and intervention project workbook for population with high risk of stroke”^35^. To be eligible, participants had to be aged 50 to 75 years and not have regularly exercised for at least one year (regular exercise was defined as one exercise that is practised for 30 minutes or longer each time at least three times per week for more than 3 months). Participants were excluded if they suffered from severe cerebrovascular diseases, musculoskeletal system diseases or other sport contraindications or if they had a history of stroke or a communication disorder.

The trial was advertised by posters or leaflets or by establishing a free clinic at the community centres. The community adults who were interested in this trial were assessed by two trained research assistants in the lounge room based on the eligibility criteria, and they were informed regarding the overall design of this study. If the subjects were eligible and agreed to participate, their baseline measurements were acquired after signing the written informed consent.

### Practiced the Baduanjin exercise and practiced Baduanjin form

Participants in the Baduanjin training group gathered in their community centres and practised the Baduanjin exercise that originated from the “Health Qigong Baduanjin Standard” established by the State Sports General Administration in 2003^36^. Two certified instructors instructed and supervised their training. The training programme was comprised of 60 minutes per session with five sessions per week for 12 weeks. Each session consisted of 45 minutes of Baduanjin form, a 10-minute warm-up and a 5-minute cool-down. Two research assistants managed the training spot and recorded the participants’ attendance. The participants in the control group were informed to maintain their usual physical activity during the 12-week intervention period.

Following the 12-week intervention period, participants of both the Baduanjin training and control groups entered an additional 12-week unsupervised follow-up period in which no additional exercise intervention was administered to any participant.

In addition, all participants were asked to keep a daily diary of physical activity level during the 12-week intervention period and the additional 12-week follow-up period. The physical activity level was assessed by using self-reported form, in which the duration and intensity of physical activity for each individual in a whole day was classified into three sections including the duration of low-intensity activity (e.g. walking, drop around, walk the dog), the duration of moderate-intensity activity (e.g. brisk walking, square dances, housework), and duration of high-intensity activity (jogging, play ball, swimming).

### Measures

The primary and secondary outcomes were assessed at baseline (−2 to −1 week), the end of the intervention (13 weeks) and the end of the additional 12-week follow-up period (25 weeks). All measurements were performed by the professional operators who did not know the allocation group at the Fujian University of Traditional Chinese Medicine Subsidiary Rehabilitation Hospital.

The primary outcomes of this trial were cerebrovascular function, consisting of cerebral haemodynamics and cerebrovascular elasticity. Cerebral haemodynamics was expressed by the maximum blood flow velocity (BFV_max_), minimum blood flow velocity (BFV_min_) and mean blood flow velocity (BFV_mean_) parameters, whereas cerebrovascular elasticity was expressed using the vascular pulsatility index (PI, calculated using following formula: (peak systolic velocity - end-diastolic velocity)/mean flow velocity)) and the vascular resistance index (RI, calculated by following formula: (peak systolic velocity – end-diastolic velocity)/end-diastolic velocity)). Those variables were measured in nine main cerebral arteries, including the bilateral vertebral artery (VA), bilateral middle cerebral artery (MCA), bilateral anterior cerebral artery (ACA), bilateral posterior cerebral artery (PCA) and basilar artery (BA), using a colour Doppler ultrasound imaging device (Philips, product type: IU22).

The secondary outcomes were cardiac structure and cardiac function, static pulmonary function, traditional risk factors of CVD and psychological outcomes. Cardiac structure and cardiac function were measured using the colour Doppler ultrasound imaging device (product type: SIEMENS Acuson X300). Static lung function was measured using cardiopulmonary function instruments (JAEGER, Germany, product type: OXYCON PRO PC). Traditional risk factors of CVD include blood pressure, heart rate, plasma indicators and anthropometric measurements. The blood pressure was measured manually by the professional staff at the hospital. The plasma indicators of CVD include blood lipid and fasting blood glucose, which were measured quantitatively using an automatic analyser according to the specifications of the fasting period using kit (Rongshen, Shanghai) and enzymatic method (NSA-400, Shenyang). The anthropometric measurements include body mass index (BMI, calculated by weight (kg)/height (m^2^)), waist circumference, hip circumference and waist-to-hip ratio (waist circumference (cm)/hip circumference (cm)), which were measured using a body scale for BMI and a metric ruler for waist circumference and hip circumference. The related psychological outcomes, including mood, self-confidence and self-esteem, quality of life and sleep quality, were assessed using the Brief Profile of Mood States (BPOMS), the Rosenberg Self-Esteem Scale, the Chinese version of the Social Functioning 36 (SF-36) scale and the Pittsburgh Sleep Quality Index (PSQI), respectively.

### Sample Size and Statistical Analysis

A priori sample size calculation was based on an anticipated 10% improvement in cerebral haemodynamic parameters among participants undergoing the 12-week Baduanjin training compared to those in the control group. 85 participants per group was determined a priori based on a 1:1 treatment allocation, considering the target effect size detected, a significance level of 0.05 (type I error), an analysis power of 90% (type II error of 0.10), and a maximum loss to follow-up of 15%^34^.

All statistical analyses were conducted by a blinded statistician using the IBM SPSS 21.0 (IBM Inc., Chicago, IL, USA) statistical software package. A two-sided *p* value of less than 0.05 was considered significantly different. Baseline characteristics between the two groups were analysed using the *t* test or the Mann-Whitney test for continuous variables and Pearson’s χ^2^ test for categorical variables. Using the linear mixed models based on the intention-to-treat (ITT) principle, a multiple analysis assessed the changes in cerebrovascular function, cardiopulmonary function, plasma traditional risk indicators of CVD, anthropometric measurements, quality of life, happiness, mood and sleep quality from baseline to the endpoint of the intervention or from baseline to the endpoint of follow-up between the Baduanjin group and the control group. The main between-group effect (2 levels: Baduanjin vs control) and interaction effect for group and time point (3 levels: baseline, 12 weeks and 24 weeks) were treated as fixed effects with time and a repeated fixed effect, whereas the participants were random variables. The baseline data and comparison group were used as the reference variable. A fully unstructured variance-covariance matrix was assumed for the error terms. Missing data were imputed using the multiple imputation method based on the Fully Conditional Specification (FCS) algorithm.

## Results

### Participant Characteristics at Baseline

**Figure 1:**
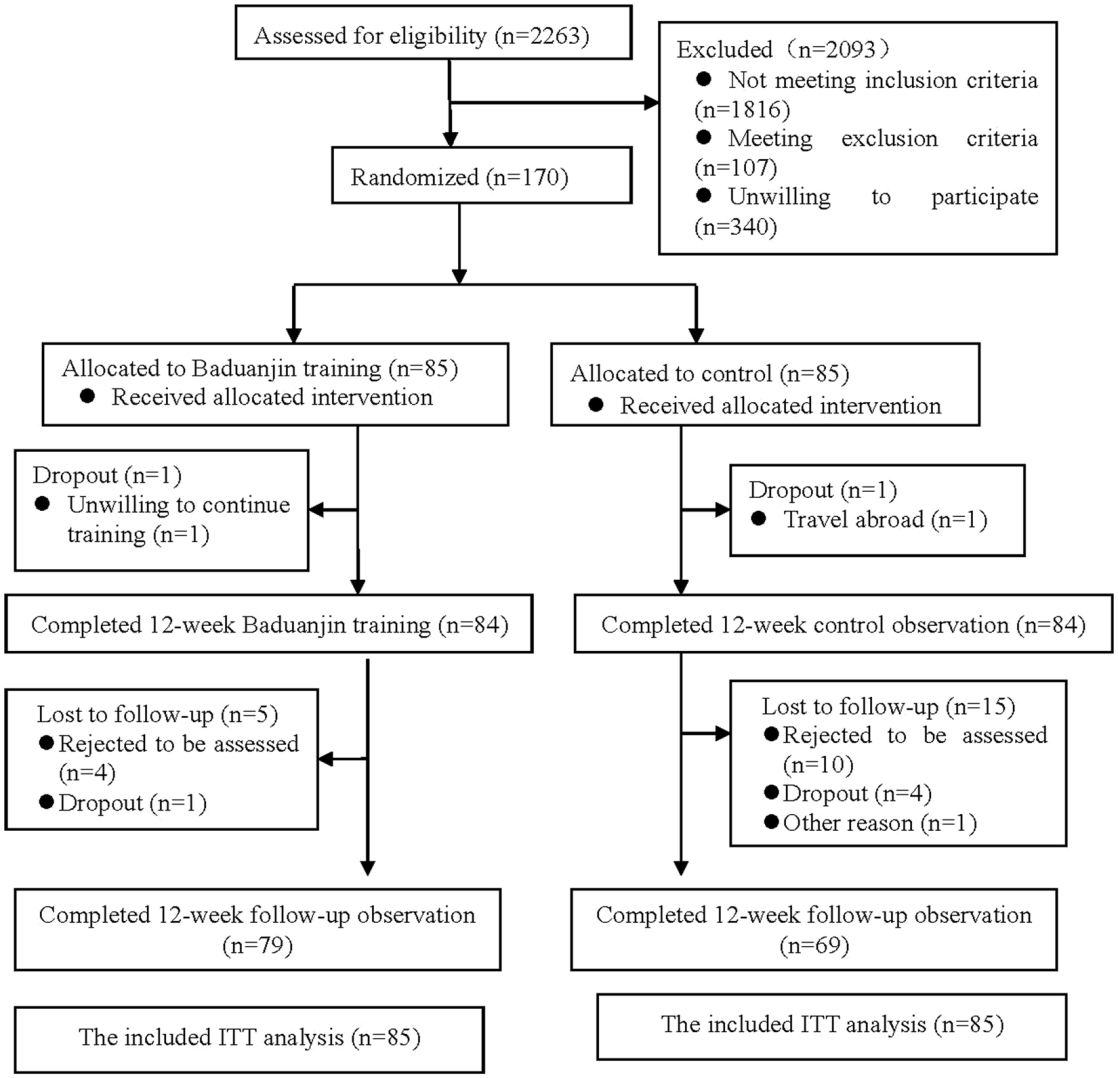
Participants flow through the study reported in a CONSORT diagram.

Figure 1 outlines the flow of participants during the trial. Recruitment started on 1 November 2013 and was completed on 28 February 2014. A total of 2,263 older community adults were assessed for eligibility, and 2,093 individuals were excluded because of conflicts with the eligibility criteria or because they were unwilling to participate. In total, 170 eligible participants were randomized into either the Baduanjin training group or the usual physical activity control group with an equal allocation rate. The baseline characteristics were similar between groups (Table 1).

**Table 1:** Baseline demographic characteristics of participants between Baduanjin group and control group.

Variables	Control group (n = 85)	Baduanjin group(n = 85)
Age, years (mean ± SD)	59.75 ± 6.34	60.53 ± 6.29
**Gender (n, %)**		
Male	31 (36.5)	30 (35.3)
Female	54 (63.5)	55 (64.7)
**Overweight** ^*^ **(n, %)**		
Yes	56 (65.9)	50 (58.8)
No	29 (34.1)	35 (41.2)
**Smoking (n, %)**		
Yes	15 (17.6)	8 (9.4)
No	70 (82.4)	77 (90.6)
**Diabetes (n, %)**		
Yes	17 (20)	17 (20)
No	68 (80)	68 (80)
**Hypertension** ^*^ **(n, %)**		
Yes	43 (50.6)	46 (54.1)
No	42 (49.4)	39 (45.9)
**Dyslipidemia** ^*^ **(n, %)**		
Yes	67 (78.8)	62 (72.9)
No	18 (21.2)	23 (27.1)
**Fibrillation atrial(n, %)**		
Yes	1 (1.2)	4 (4.7)
No	84 (98.8)	81 (95.3)
**Family history of stroke (n, %)**		
Yes	32 (37.6)	30 (35.3)
No	53 (62.4)	55 (64.7)
**Transient ischemic attack(n, %)**		
Yes	18 (21.2)	18 (21.2)
No	67 (78.8)	67 (78.8)

*Definition: Overweight (BMI ≥ 25); hypertension (blood pressure ≥ 140/90 mmHg); dyslipidemia (TC ≥ 6.22 mmol/L, or TG ≥ 2.26 mmol/L, or LDL-C ≥ 4.14 mmol/L, or HDL-C ≤ 1.04 mmol/L).

### Compliance

Of the 170 participants who completed the baseline assessment, 168 (98.8%) performed the post-intervention evaluation, and 148 (87.1%) completed the 12-week follow-up. The dropout rate was significantly higher in the control group than in the Baduanjin training group according to Pearson’s Chi-square test (χ^2^ = 5.221, *P* = 0.04), but no significant difference was found in the baseline characteristics except for fibrillation atrial in between the subjects who dropped out in the study and the subjects who completed the whole study.

### Daily Physical Activity During Study Period

Table 2 presents the daily physical activity between Baduanjin training group and control group during study period. The average duration of low intensity activity was not significantly different between the two groups during the study period. The average duration of moderate intensity activity in the Baduanjin exercise group was higher than that for the control group. No high intensity physical activity was reported during the study period.

**Table 2:** Comparison of the daily physical activities between groups during study period (hour, mean ± standard deviation).

Study period	Intensity	Control group		Baduanjin group		T(Z)	*P* value
		n	M ± SD	n	M ± SD		
Intervention period	low intensity	84	3.62 ± 1.69	84	3.61 ± 1.50	0.048	0.962
	Moderate intensity		4.65 ± 1.88		5.81 ± 1.86	−4.064	<0.001
	High intensity		0		0	/	/
Follow-up period	low intensity	69	3.54 ± 1.49	79	3.50 ± 1.70	0.168	0.867
	Moderate intensity		4.75 ± 1.81		5.54 ± 1.92	−2.754	0.007
	High intensity		0		0	/	/

M: mean; SD: standard deviation.

### Cerebrovascular Haemodynamic Parameters

Table 3 presents the changes in haemodynamic parameters, including the maximum, minimum and mean blood flow velocities (BFV_max_, BFV_min_ and BFV_mean_) and the PI and RI in the cerebral basilar arteries (BAs), bilateral vertebral arteries (LVA and RVA), bilateral anterior, and middle and posterior arteries (LAA, RAA, LMA, RMA, LPA and RPA) at baseline, at 12 weeks post-intervention and after an additional 12 weeks of follow-up. There was no significant difference in any parameters between the groups at baseline. Linear mixed models with repeated measures showed a significant interaction effect of group (intervention) by time in the PI and RI parameters for all the measured cerebrovascular arteries; in BFV_max_ for BA, RVA and LAA; and in BFV_min_ and BFV_mean_ for the BA, LVA and RVA. A significant main effect of group in RI for BA; in BFV_max_ for LVA, and in BFV_mean_ for RMA was observed. These results indicated a significant improvement in those cerebrovascular haemodynamic parameters in the Baduanjin exercise group compared with the control group. After the 12-week intervention, a significant difference between the groups was found in almost all parameters except for the RI in the BA and RMA, BFV_mean_ in the RVA; BFV_max_ in the RMA; and BFV_max_, BFV_mix_ and BFV_min_ in the LAA, LMA and RPA. A significant difference between the groups was also determined in all the measured parameters except BFV_max_ in the RPA, BA and LVA; BAF_mean_ and BAF_min_ in the RAA; and five haemodynamic parameters (BFV_max_, BFV_min_, BFV_mean_, PI and RI) in the LPA after the additional 12-week follow-up period. Furthermore, the differences showed that those parameters were significantly lower in the Baduanjin intervention group than in the control group (Table 3).

**Table 3:** Mean haemodynamic parameters of five cerebrovascular arteries at all time points.

Variables	Time points	Control		Baduanjin		Mean difference between comparison group (95% CI)	*P* value	Mixed linear model	
		N	Mean ± SE	N	Mean ± SE			*P*-value (between-groups)	*P*-value (Time × Group)
**Basilar artery (BA)**									
**BFV**_max_, cm/s	Baseline	85	69.17 ± 3.01	85	67.67 ± 1.58	1.49 (−4.69–7.67)	0.660		
	12-weeks	85	67.96 ± 3.07	85	60.50 ± 1.39	7.46 (0.79–14.13)	0.028		
	24-weeks	85	64.95 ± 2.69	85	59.27 ± 1.47	5.69 (−0.36–11.73)	0.065	0.111	0.003
**BFV**_min_, cm/s	Baseline	85	63.42 ± 2.76	85	62.33 ± 1.50	1.19 (−4.99–7.38)	0.704		
	12-weeks	85	63.28 ± 3.01	85	55.84 ± 1.33	7.44 (0.95–13.93)	0.025		
	24-weeks	85	61.26 ± 2.50	85	55.03 ± 1.41	6.23 (0.56–11.90)	0.031	0.088	0.001
**BFV**_mean_, cm/s	Baseline	85	66.38 ± 2.86	85	65.05 ± 1.53	1.34 (−5.08–7.75)	0.681		
	12-weeks	85	65.80 ± 3.04	85	58.11 ± 1.35	7.69 (1.12–14.27)	0.022		
	24-weeks	85	63.42 ± 2.54	85	57.14 ± 1.47	6.29 (0.50–12.07)	0.033	0.086	0.001
**PI**	Baseline	85	1.11 ± 0.22	85	1.14 ± 0.22	−0.04 (−0.10–0.03)	0.265		
	12-weeks	85	1.17 ± 0.03	85	1.08 ± 0.02	0.08 (0.02–0.15)	0.01		
	24-weeks	85	1.14 ± 0.04	85	1.04 ± 0.02	0.10 (0.017–0.188)	0.019	0.060	<0.001
**RI**	Baseline	85	0.63 ± 0.01	85	0.64 ± 0.01	−0.01 (−0.03–0.01)	0.291		
	12-weeks	85	0.65 ± 0.01	85	0.63 ± 0.01	0.03 (0.01–0.04)	0.12		
	24-weeks	85	0.64 ± 0.01	85	0.61 ± 0.01	0.037 (0.01–0.06)	0.01	0.045	<0.001
**Left vertebral artery** (**LVA)**									
**BFV**_max_, cm/s	Baseline	85	64.70 ± 4.47	85	60.47 ± 1.74	4.23 (−5.23–13.70)	0.379		
	12-weeks	85	61.31 ± 2.24	85	54.79 ± 1.41	6.51 (1.29–11.74)	0.015		
	24-weeks	85	59.19 ± 1.95	85	54.59 ± 1.51	4.60 (−0.27–9.46)	0.064	0.036	0.563
**BFV**_min_, cm/s	Baseline	85	55.60 ± 1.77	85	55.46 ± 1.68	0.14 (−4.68–4.96)	0.955		
	12-weeks	85	56.13 ± 2.18	85	50.45 ± 1.36	5.68 (0.61–10.75)	0.028		
	24-weeks	85	54.83 ± 1.66	85	50.12 ± 1.41	4.71 (0.42–9.00)	0.032	0.102	0.007
**BFV**_mean_, cm/s	Baseline	85	58.02 ± 1.82	85	57.92 ± 1.70	0.10 (−4.82–5.02)	0.968		
	12-weeks	85	58.96 ± 2.21	85	52.84 ± 1.40	6.11 (0.96–11.28)	0.02		
	24-weeks	85	57.44 ± 1.69	85	52.42 ± 1.45	5.02 (0.62–9.42)	0.026	0.089	0.003
**PI**	Baseline	85	1.10 ± 0.024	85	1.16 ± 0.022	−0.06 (−0.12–0.07)	0.079		
	12-weeks	85	1.16 ± 0.02	85	1.09 ± 0.02	0.073 (0.014–0.132)	0.016		
	24-weeks	85	1.15 ± 0.02	85	1.05 ± 0.02	0.10 (0.04–0.16)	0.001	0.095	<0.001
**RI**	Baseline	85	0.63 ± 0.008	85	0.65 ± 0.007	−0.02 (−0.04–0.02)	0.069		
	12-weeks	85	0.65 ± 0.007	85	0.63 ± 0.007	0.025 (0.005–0.043)	0.013		
	24-weeks	85	0.65 ± 0.008	85	0.61 ± 0.007	0.034 (0.013–0.055)	0.001	0.087	<0.001
**Right vertebral artery** (**RVA)**									
**BFV**_max_, cm/s	Baseline	85	60.92 ± 1.89	85	59.89 ± 1.69	1.03 (−3.98–6.03)	0.686		
	12-weeks	85	60.43 ± 2.16	85	55.25 ± 1.34	5.18 (0.10–10.27)	0.046		
	24-weeks	85	60.34 ± 1.78	85	54.13 ± 1.46	6.21 (1.66–10.76)	0.008	0.056	0.037
**BFV**_min_, cm/s	Baseline	85	55.31 ± 1.76	85	54.70 ± 1.67	0.61 (−4.18–5.40)	0.802		
	12-weeks	85	55.25 ± 2.11	85	50.32 ± 1.34	4.93 (0.01–9.86)	0.05		
	24-weeks	85	55.49 ± 1.70	85	49.79 ± 1.43	5.70 (1.31–10.08)	0.011	0.074	0.027
**BFV**_mean_, cm/s	Baseline	85	58.11 ± 1.83	85	57.35 ± 1.69	0.76 (−4.16–5.68)	0.761		
	12-weeks	85	57.81 ± 2.13	85	53.13 ± 1.37	4.68 (−0.32–9.67)	0.066		
	24-weeks	85	57.83 ± 1.76	85	52.04 ± 1.46	5.78 (1.27–10.30)	0.012	0.083	0.039
**PI**	Baseline	85	1.10 ± 0.023	85	1.18 ± 0.02	−0.08 (−0.14–0.02)	0.015		
	12-weeks	85	1.20 ± 0.023	85	1.10 ± 0.02	0.11 (0.05–0.17)	0.001		
	24-weeks	85	1.15 ± 0.03	85	1.01 ± 0.02	0.14 (0.06–0.21)	0.001	0.021	<0.001
**RI**	Baseline	85	0.63 ± 0.008	85	0.65 ± 0.007	−0.02(−0.04–0.01)	0.016		
	12-weeks	85	0.66 ± 0.007	85	0.63 ± 0.006	0.03 (0.013–0.053)	0.001		
	24-weeks	85	0.64 ± 0.009	85	0.60 ± 0.007	0.04 (0.02–0.07)	<0.001	0.022	<0.001
**Left anterior artery** (**LAA)**									
**BFV**_max_, cm/s	Baseline	69	85.74 ± 2.84	69	89.58 ± 2.56	−3.84 (−11.40–3.72)	0.317		
	12-weeks	69	87.01 ± 2.31	69	81.80 ± 2.07	5.21 (−0.92–11.34)	0.095		
	24-weeks	69	85.55 ± 2.03	69	79.88 ± 1.71	5.67 (0.42–10.93)	0.035	0.319	0.048
**BFV**_min_, cm/s	Baseline	69	79.58 ± 2.80	69	82.49 ± 2.55	−2.91 (−10.40–4.58)	0.443		
	12-weeks	69	81.45 ± 2.21	69	75.98 ± 1.97	5.47 (−0.39–11.33)	0.067		
	24-weeks	69	78.68 ± 1.79	69	73.80 ± 1.67	4.88 (0.04–9.72)	0.048	0.286	0.073
**BFV**_mean_, cm/s	Baseline	69	82.64 ± 2.80	69	86.10 ± 2.49	−3.46 (−10.87–3.94)	0.357		
	12-weeks	69	84.07 ± 2.23	69	78.82 ± 1.99	5.24 (−0.66–11.15)	0.067		
	24-weeks	69	82.54 ± 1.90	69	76.79 ± 1.71	5.76 (0.70–10.81)	0.026	0.275	0.052
**PI**	Baseline	69	1.03 ± 0.025	69	1.06 ± 0.019	−0.03 (−0.09–0.03)	0.363		
	12-weeks	69	1.09 ± 0.022	69	1.00 ± 0.018	0.09 (0.036–0.148)	0.001		
	24-weeks	69	1.06 ± 0.029	69	0.91 ± 0.021	0.15 (0.077–0.219)	<0.001	0.005	<0.001
**RI**	Baseline	69	0.61 ± 0.009	69	0.62 ± 0.006	−0.01 (−0.03–0.01)	0.371		
	12-weeks	69	0.63 ± 0.007	69	0.60 ± 0.006	0.03 (0.012–0.049)	0.001		
	24-weeks	69	0.62 ± 0.01	69	0.56 ± 0.008	0.05 (0.026–0.079)	<0.001	0.005	<0.001
**Right anterior artery** (**RAA)**									
**BFV**_max_, cm/s	Baseline	58	88.88 ± 3.19	64	87.55 ± 2.52	1.33 (−6.64–9.31)	0.741		
	12-weeks	58	91.93 ± 2.54	64	83.59 ± 2.10	8.35 (1.86–14.83)	0.012		
	24-weeks	58	87.51 ± 1.90	64	83.14 ± 1.66	4.37 (−0.60–9.34)	0.085	0.069	0.135
**BFV**_min_, cm/s	Baseline	58	83.19 ± 3.14	64	81.44 ± 2.50	1.75 (−6.12–9.62)	0.660		
	12-weeks	58	86.19 ± 2.47	64	78.29 ± 2.09	7.91 (1.54–14.27)	0.015		
	24-weeks	58	81.92 ± 1.89	64	77.45 ± 1.68	4.46 (−0.52–9.44)	0.079	0.065	0.190
**BFV**_mean_, cm/s	Baseline	58	85.78 ± 3.16	64	84.80 ± 2.51	0.98 (−6.94–8.90)	0.807		
	12-weeks	58	88.91 ± 2.46	64	80.95 ± 2.11	7.96 (1.57–14.35)	0.015		
	24-weeks	58	84.88 ± 1.94	64	80.34 ± 1.67	4.54 (−0.50–9.58)	0.077	0.078	0.144
**PI**	Baseline	58	1.06 ± 0.028	64	1.06 ± 0.020	−0.001 (−0.07–0.06)	0.979		
	12-weeks	58	1.11 ± 0.026	64	1.01 ± 0.019	0.10 (0.04–0.167)	0.002		
	24-weeks	58	1.00 ± 0.029	64	0.91 ± 0.018	0.09 (0.03–0.161)	0.005	0.01	0.009
**RI**	Baseline	58	0.61 ± 0.009	64	0.62 ± 0.007	−0.01 (−0.03–0.019)	0.712		
	12-weeks	58	0.64 ± 0.009	64	0.60 ± 0.007	0.035 (0.012–0.06)	0.003		
	24-weeks	58	0.60 ± 0.01	64	0.56 ± 0.007	0.04 (0.01–0.06)	0.006	0.018	0.004
**Left middle artery** (**LMA)**									
**BFV**_max_, cm/s	Baseline	68	99.18 ± 3.82	72	97.07 ± 3.18	2.11 (−7.69–11.90)	0.671		
	12-weeks	68	98.15 ± 3.13	72	92.27 ± 2.83	5.89 (−2.43–14.21)	0.164		
	24-weeks	68	96.79 ± 2.95	72	89.28 ± 2.42	7.51 (0.01–15.02)	0.05	0.159	0.503
**BFV**_min_, cm/s	Baseline	68	93.07 ± 3.72	72	89.39 ± 3.00	3.69 (−5.72–13.09)	0.440		
	12-weeks	68	91.68 ± 2.99	72	86.05 ± 2.79	5.63 (−2.45–13.71)	0.170		
	24-weeks	68	90.66 ± 2.77	72	82.91 ± 2.28	7.75 (0.69–14.82)	0.032	0.109	0.660
**BFV**_mean_, cm/s	Baseline	68	95.69 ± 3.71	72	93.10 ± 3.02	2.59 (−6.81–11.99)	0.586		
	12-weeks	68	95.51 ± 3.08	72	89.30 ± 2.81	6.21 (−2.02–14.44)	0.138		
	24-weeks	68	94.06 ± 2.87	72	86.33 ± 2.37	7.74 (0.41–15.06)	0.039	0.125	0.498
**PI**	Baseline	68	0.96 ± 0.026	72	0.99 ± 0.019	−0.03 (−0.09–0.03)	0.347		
	12-weeks	68	1.04 ± 0.023	72	0.96 ± 0.016	0.084 (0.029–0.139)	0.003		
	24-weeks	68	0.98 ± 0.027	72	0.88 ± 0.017	0.095 (0.033–0.157)	0.003	0.047	<0.001
**RI**	Baseline	68	0.58 ± 0.009	72	0.59 ± 0.007	−0.017 (−0.04–0.006)	0.146		
	12-weeks	68	0.61 ± 0.008	72	0.58 ± 0.006	0.029 (0.01–0.047)	0.003		
	24-weeks	68	0.59 ± 0.009	72	0.55 ± 0.007	0.033 (0.01–0.056)	0.005	0.088	<0.001
**Right middle artery** (**RMA)**									
**BFV**_max_, cm/s	Baseline	85	95.51 ± 2.36	85	92.37 ± 2.25	3.14 (−3.29–9.57)	0.337		
	12-weeks	85	97.11 ± 2.17	85	90.58 ± 1.91	6.53 (0.82–12.24)	0.025		
	24-weeks	85	94.20 ± 1.81	85	86.21 ± 1.63	7.99 (3.18–12.81)	0.001	0.012	0.249
**BFV**_min_, cm/s	Baseline	85	88.75 ± 2.34	85	85.80 ± 2.22	2.95 (−3.42–9.32)	0.361		
	12-weeks	85	88.98 ± 2.04	85	84.25 ± 1.91	4.73 (−0.78–10.25)	0.092		
	24-weeks	85	86.34 ± 1.80	85	79.64 ± 1.58	6.71 (1.99–11.43)	0.006	0.022	0.446
**BFV**_mean_, cm/s	Baseline	85	91.91 ± 2.35	85	88.80 ± 2.23	3.11 (−3.29–9.51)	0.339		
	12-weeks	85	93.98 ± 2.11	85	87.10 ± 1.92	6.88 (1.25–12.51)	0.017		
	24-weeks	85	90.35 ± 1.80	85	83.74 ± 1.62	6.61 (1.83–11.40)	0.007	0.017	0.360
**PI**	Baseline	85	0.94 ± 0.022	85	1.00 ± 0.021	−0.06 (−0.113–0.005)	0.071		
	12-weeks	85	1.03 ± 0.023	85	0.96 ± 0.019	0.06 (0.004–0.122)	0.038		
	24-weeks	85	0.94 ± 0.021	85	0.87 ± 0.021	0.07 (0.013–0.129)	0.017	0.057	<0.001
**RI**	Baseline	85	0.57 ± 0.009	85	0.59 ± 0.009	−0.02 (−0.045–0.004)	0.100		
	12-weeks	85	0.60 ± 0.01	85	0.58 ± 0.008	0.017 (−0.01–0.04)	0.182		
	24-weeks	85	0.58 ± 0.009	85	0.55 ± 0.009	0.027 (0.002–0.051)	0.032	0.066	<0.001
**Left posterior artery** (**LPA)**									
**BFV**_max_, cm/s	Baseline	68	53.66 ± 1.34	67	54.61 ± 1.37	−0.95 (−4.75–2.85)	0.621		
	12-weeks	68	54.36 ± 1.29	67	50.71 ± 1.19	3.65 (0.16–7.14)	0.041		
	24-weeks	68	54.32 ± 1.12	67	51.50 ± 1.30	2.82 (−0.57–6.22)	0.103	0.133	0.140
**BFV**_min_, cm/s	Baseline	68	48.71 ± 1.31	67	49.15 ± 1.38	−0.44 (−4.21–3.32)	0.816		
	12-weeks	68	49.63 ± 1.25	67	46.02 ± 1.08	3.61 (0.34–6.88)	0.031		
	24-weeks	68	49.38 ± 0.97	67	46.51 ± 1.21	2.87 (−0.19–5.94)	0.066	0.088	0.182
**BFV**_mean_, cm/s	Baseline	68	51.24 ± 1.32	67	51.97 ± 1.38	−0.74 (−4.51–3.04)	0.701		
	12-weeks	68	51.94 ± 1.25	67	48.59 ± 1.17	3.35 (−0.05–6.74)	0.053		
	24-weeks	68	52.02 ± 1.01	67	49.12 ± 1.17	2.90 (−0.15–5.95)	0.062	0.122	0.177
**PI**	Baseline	68	1.03 ± 0.023	67	1.06 ± 0.022	−0.03 (−0.09–0.032)	0.329		
	12-weeks	68	1.13 ± 0.022	67	1.03 ± 0.019	0.092 (0.034–0.151)	0.002		
	24-weeks	68	1.02 ± 0.029	67	0.95 ± 0.022	0.07 (−0.001–0.141)	0.055	0.074	0.003
**RI**	Baseline	68	0.61 ± 0.008	67	0.62 ± 0.007	−0.01 (−0.03–0.01)	0.360		
	12-weeks	68	0.64 ± 0.007	67	0.61 ± 0.007	0.03 (0.011–0.050)	0.003		
	24-weeks	68	0.60 ± 0.01	67	0.58 ± 0.008	0.023 (−0.002–0.048)	0.071	0.08	0.006
**Right posterior artery** (**RPA)**									
**BFV**_max_, cm/s	Baseline	56	49.55 ± 1.57	64	51.55 ± 1.59	−1.99 (−6.44–2.45)	0.376		
	12-weeks	56	51.71 ± 1.28	64	49.90 ± 1.36	1.80 (−1.91–5.52)	0.339		
	24-weeks	56	53.16 ± 1.29	64	50.51 ± 1.16	2.66 (−0.78–6.09)	0.128	0.547	0.200
**BFV**_min_, cm/s	Baseline	56	45.04 ± 1.53	64	46.39 ± 1.53	−1.36 (−5.67–2.96)	0.535		
	12-weeks	56	46.92 ± 1.20	64	44.85 ± 1.31	2.07 (−1.48–5.62)	0.251		
	24-weeks	56	48.75 ± 1.29	64	45.32 ± 1.06	3.43 (0.16–6.71)	0.04	0.298	0.179
**BFV**_mean_, cm/s	Baseline	56	47.23 ± 1.55	64	49.28 ± 1.57	−2.05 (−6.44–2.24)	0.357		
	12-weeks	56	49.66 ± 1.28	64	47.46 ± 1.30	2.20 (−1.44–5.83)	0.233		
	24-weeks	56	50.90 ± 1.25	64	47.88 ± 1.08	3.03 (−0.23–6.28)	0.068	0.429	0.134
**PI**	Baseline	56	1.04 ± 0.025	64	1.09 ± 0.023	−0.05 (−0.117–0.015)	0.128		
	12-weeks	56	1.12 ± 0.024	64	1.01 ± 0.020	0.11 (0.05–0.174)	<0.001		
	24-weeks	56	1.02 ± 0.028	64	0.87 ± 0.023	0.14 (0.07–0.21)	<0.001	0.005	<0.001
**RI**	Baseline	56	0.61 ± 0.008	64	0.63 ± 0.008	−0.02 (−0.04–0.003)	0.09		
	12-weeks	56	0.64 ± 0.008	64	0.60 ± 0.007	0.04 (0.018–0.058)	<0.001		
	24-weeks	56	0.60 ± 0.01	64	0.55 ± 0.009	0.05 (0.028–0.08)	<0.001	0.004	<0.001

Abbreviations: BFV, blood flow velocity; PI, pulsatility index; RI, resistance index.

### Cardiac Structure, Cardiac Function and Static Pulmonary Function

We found no significant differences in all parameters of cardiac structure and function and static pulmonary function, including left atrial diameter, right ventricular diameter, left ventricular ejection fraction, minute ventilation volume, vital capacity and maximal voluntary ventilation, between the Baduanjin exercise group and the control group, whereas a significant interaction of group by time was also not identified by the mixed linear model analysis (Table 4).

**Table 4:** Mean cardiopulmonary function outcomes at all time points (Mean ± SE).

Variables	Time points	Control		Baduanjin		Mean difference (95% CI)	P value	Mixed linear model	
								*P*-value (between-groups)	*P*-value (Time × Group)
		n	Mean ± SE	n	Mean ± SE				
MVV (L/min)	Baseline	85	11.64 ± 0.59	85	11.45 ± 0.53	0.18 (−1.39~1.76)	0.820		
	12-weeks	85	12.55 ± 0.68	85	12.62 ± 0.68	−0.06 (−1.96~1.83)	0.948		
	24-weeks	85	11.05 ± 1.04	85	12.20 ± 0.60	−1.16 (−3.53~1.21)	0.337	0.611	0.637
VC (L)	Baseline	85	2.90 ± 0.08	85	2.93 ± 0.11	−0.03 (−0.31~0.23)	0.770		
	12-weeks	85	3.03 ± 0.07	85	3.09 ± 0.07	−0.06 (−0.26~0.14)	0.546		
	24-weeks	85	3.14 ± 0.09	85	3.15 ± 0.08	−0.01 (−0.24~0.23)	0.955	0.735	0.813
VV_max_ (L/min)	Baseline	85	84.44 ± 2.77	85	86.35 ± 3.14	−1.91 (−10.18~6.37)	0.650		
	12-weeks	85	85.87 ± 2.35	85	89.03 ± 2.83	−3.16 (−10.43~4.11)	0.167		
	24-weeks	85	94.14 ± 2.29	85	100.62 ± 2.33	−6.48 (−12.93~−0.03)	0.049	0.203	0.605
TV (L)	Baseline	85	0.59 ± 0.02	85	0.61 ± 0.03	−0.02 (−0.08~0.04)	0.522		
	12-weeks	85	0.75 ± 0.04	85	0.75 ± 0.03	−0.006 (−0.11~0.09)	0.907		
	24-weeks	85	0.68 ± 0.03	85	0.64 ± 0.03	0.04 (−0.05~0.13)	0.372	0.894	0.461
FEV1 (L)	Baseline	85	2.38 ± 0.06	85	2.46 ± 0.06	−0.08 (−0.24~0.080	0.306		
	12-weeks	85	2.39 ± 0.05	85	2.50 ± 0.06	−0.11 (−0.25~0.04)	0.154		
	24-weeks	85	2.47 ± 0.05	85	2.48 ± 0.05	−0.01 (−0.16~0.14)	0.888	0.355	0.098
IC (L)	Baseline	85	2.32 ± 0.07	85	2.26 ± 0.09	0.05 (−0.18~0.28)	0.659		
	12-weeks	85	2.45 ± 0.07	85	2.50 ± 0.05	−0.05 (−0.21~0.12)	0.596		
	24-weeks	85	2.46 ± 0.08	85	2.41 ± 0.07	0.05 (−0.17~0.26)	0.651	0.823	0.512
FEV1/FVC (%)	Baseline	85	89.01 ± 0.96	85	89.67 ± 2.01	−0.67 (−5.08~3.74)	0.764		
	12-weeks	85	82.67 ± 0.73	85	84.27 ± 0.67	−1.60 (−3.57~0.37)	0.111		
	24-weeks	85	83.71 ± 1.15	85	84.06 ± 0.88	−0.35 (−3.20~2.49)	0.808	0.437	0.703
AD (mm)	Baseline	85	2.83 ± 0.03	85	2.80 ± 0.30	0.03 (−0.07~0.12)	0.570		
	12-weeks	85	2.90 ± 0.04	85	2.92 ± 0.04	−0.02 (−0.14~0.08)	0.658		
	24-weeks	85	2.89 ± 0.04	85	2.86 ± 0.03	0.03 (−0.07~0.13)	0.561	0.792	0.534
LAD (cm)	Baseline	85	3.18 ± 0.04	85	3.22 ± 0.04	−0.04 (−0.16~0.08)	0.487		
	12-weeks	85	3.22 ± 0.04	85	3.29 ± 0.04	−0.07 (−0.18~0.04)	0.210		
	24-weeks	85	3.11 ± 0.04	85	3.11 ± 0.05	−0.005 (−0.14~0.13)	0.940	0.414	0.642
IVST (cm)	Baseline	85	1.02 ± 0.01	85	1.01 ± 0.01	0.01 (−0.02~0.05)	0.360		
	12-weeks	85	0.98 ± 0.01	85	0.98 ± 0.01	0.00 (−0.03~0.03)	0.955		
	24-weeks	85	0.98 ± 0.01	85	0.97 ± 0.01	0.01 (−0.02~0.04)	0.539	0.480	0.739
LVPWT	Baseline	85	0.94 ± 0.01	85	0.95 ± 0.01	−0.01 (−0.03~0.02)	0.666		
	12-weeks	85	0.94 ± 0.01	85	0.93 ± 0.01	0.01 (−0.01~0.04)	0.236		
	24-weeks	85	0.93 ± 0.01	85	0.92 ± 0.01	0.01 (−0.03~0.03)	0.825	0.638	0.422
RVD (cm)	Baseline	85	3.00 ± 0.03	85	2.97 ± 0.03	0.03 (−0.07~0.13)	0.541		
	12-weeks	85	2.90 ± 0.03	85	2.99 ± 0.03	−0.09 (−0.20~0.01)	0.068		
	24-weeks	85	2.86 ± 0.03	85	2.82 ± 0.03	0.04 (−0.07~0.14)	0.482	0.756	0.09
MPAD	Baseline	85	2.05 ± 0.02	85	2.04 ± 0.02	0.01 (−0.06~0.08)	0.812		
	12-weeks	85	2.08 ± 0.02	85	2.09 ± 0.02	−0.01 (−0.06~0.05)	0.815		
	24-weeks	85	2.07 ± 0.02	85	2.10 ± 0.02	−0.03 (−0.09~0.04)	0.454	0.706	0.795
LVEF (%)	Baseline	85	61.92 ± 0.41	85	62.29 ± 0.47	−0.37 (0.63~1.61)	0.548		
	12-weeks	85	63.16 ± 0.39	85	63.47 ± 0.43	−0.36 (0.58~−1.51)	0.540		
	24-weeks	85	61.65 ± 0.34	85	62.37 ± 0.32	−0.98 (−1.94~−0.02)	0.046	0.125	0.617

Abbreviations: MVV: Minute ventilation volume; VC: Vital capacity; VV_max_: Maximal voluntary ventilation; TV: Tidal volume; FEV1: Forced expiratory volume in one second; IC: Inspiratory capacity; FVC: Forced vital capacity; AD: Aortic diameters; LAD: left atrial diameter; IVST: Interventricular septal thickness; LVPWT: left ventricular posterior wall thickness; RVD: right ventricular diameter; MPAD: main pulmonary artery diameter; LVEF: Left ventricular ejection fraction.

### Traditional Risk Factors of Cerebrovascular Diseases

A significant main effect of group and interaction effect of group by time in the plasma risk factors including the total cholesterol (TC), triglyceride (TG), low density lipoprotein (LDL-C), high density lipoprotein (HDL-C), fasting blood glucose (FBG) and homocysteine (Hcy) was not observed (Table 5).

**Table 5:** The traditional risk factors of cardiovascular diseases at all time points.

Variables	Time points	Control		Baduanjin		Mean difference (95% CI)	*P* value	Mixed linear model	
		n	Mean ± SE	n	Mean ± SE			*P*-value (between-groups)	*P*-value (Time × Group)
TC (mmol/L)	Baseline	85	1.57 ± 0.09	85	1.48 ± 0.08	0.09 (−1.56~0.32)	0.489		
	12-weeks	85	1.53 ± 0.09	85	1.32 ± 0.06	0.21 (0.01~0.42)	0.05		
	24-weeks	85	2.03 ± 0.13	85	1.75 ± 0.09	0.28 (−0.04~0.59)	0.09	0.088	0.315
TG (mmol/L)	Baseline	85	5.67 ± 0.14	85	5.32 ± 0.10	0.35 (0.01~0.69)	0.042		
	12-weeks	85	5.22 ± 0.12	85	5.00 ± 0.10	0.22 (−0.09~0.53)	0.162		
	24-weeks	85	5.57 ± 0.12	85	5.37 ± 0.11	0.20 (−0.13~0.53)	0.241	0.084	0.451
LDL-C (mmol/L)	Baseline	85	1.32 ± 0.03	85	1.33 ± 0.03	−0.01 (−0.10~0.08)	0.759		
	12-weeks	85	1.34 ± 0.04	85	1.34 ± 0.03	0.00 (−0.09~0.09)	0.986		
	24-weeks	85	1.41 ± 0.04	85	1.39 ± 0.03	0.02 (−0.08~0.11)	0.688	0.970	0.667
HDL-C (mmol/L)	Baseline	85	3.78 ± 0.13	85	3.43 ± 0.09	0.35 (0.03~0.65)	0.03		
	12-weeks	85	3.57 ± 0.11	85	3.37 ± 0.10	0.20 (−0.09~0.49)	0.174		
	24-weeks	85	2.70 ± 0.10	85	2.63 ± 0.09	0.07 (−0.19~0.32)	0.614	0.113	0.091
FGB (mmol/L)	Baseline	85	5.88 ± 0.21	85	5.75 ± 0.10	0.13 (−0.33~0.59)	0.584		
	12-weeks	85	5.71 ± 0.14	85	5.55 ± 0.08	0.16 (−0.16~0.48)	0.328		
	24-weeks	85	5.86 ± 0.19	85	5.71 ± 0.11	0.15 (−0.28~0.58)	0.487	0.445	0.960
Hcy (mmol/L)	Baseline	85	11.44 ± 0.64	85	12.14 ± 0.66	−0.70 (−2.52~1.12)	0.448		
	12-weeks	85	13.17 ± 0.67	85	13.07 ± 0.62	0.10 (−1.69~1.89)	0.912		
	24-weeks	85	13.21 ± 0.80	85	13.27 ± 0.62	−0.06 (−2.05~1.94)	0.958	0.804	0.240
BMI (kg/m^2^)	Baseline	85	25.25 ± 0.34	85	24.61 ± 0.35	0.64 (−0.31~1.59)	0.188		
	12-weeks	85	24.96 ± 0.34	85	24.20 ± 0.35	0.76 (−0.21~1.73)	0.124		
	24-weeks	85	24.87 ± 0.33	85	24.28 ± 0.36	0.58 (−0.38~1.55)	0.235	0.174	0.221
WC (cm)	Baseline	85	87.95 ± 0.97	85	86.93 ± 1.02	1.02 (−1.76~3.81)	0.469		
	12-weeks	85	87.85 ± 1.00	85	84.03 ± 0.92	3.82 (1.13~6.51)	0.006		
	24-weeks	85	88.67 ± 0.96	85	84.64 ± 0.98	4.02 (1.32~6.73)	0.004	0.03	<0.001
HC (cm)	Baseline	85	96.65 ± 0.71	85	96.09 ± 0.79	0.55 (−1.53~2.64)	0.602		
	12-weeks	85	96.50 ± 0.65	85	94.76 ± 0.70	1.74 (−0.15~3.62)	0.071		
	24-weeks	85	96.52 ± 0.64	85	94.63 ± 0.71	1.89 (0.00~3.79)	0.05	0.151	0.006
Waist/hip ratio (%)	Baseline	85	0.91 ± 0.006	85	0.90 ± 0.007	0.01 (−0.01~0.03)	0.385		
	12-weeks	85	0.91 ± 0.007	85	0.89 ± 0.006	0.02 (0.01~0.04)	0.012		
	24-weeks	85	0.92 ± 0.006	85	0.89 ± 0.006	0.03 (0.01~0.04)	0.007	0.034	0.033
SBP (mmHg)	Baseline	85	134.3 ± 2.04	85	135.9 ± 2.01	−1.68 (−7.33~3.97)	0.557		
	12-weeks	85	133.5 ± 1.92	85	122.2 ± 1.39	11.27 (6.59~15.96)	<0.001		
	24-weeks	85	136.0 ± 2.18	85	125.7 ± 1.68	10.37 (4.93~15.81)	<0.001	0.004	<0.001
DBP (mmHg)	Baseline	85	79.27 ± 1.20	85	79.62 ± 1.19	−0.35 (−3.69~2.99)	0.835		
	12-weeks	85	78.35 ± 1.18	85	75.26 ± 0.91	3.05 (0.08~6.02)	0.044		
	24-weeks	85	80.20 ± 1.19	85	76.57 ± 0.95	2.93 (−0.23~6.08)	0.069	0.113	0.023
HR (times/min)	Baseline	85	77.9 ± 1.0	85	78.1 ± 1.0	−0.17 (−2.97~2.64)	0.908		
	12-weeks	85	76.1 ± 1.0	85	73.6 ± 0.8	2.70 (0.15~5.25)	0.038		
	24-weeks	85	73.2 ± 0.9	85	71.9 ± 0.9	1.64 (−1.12~4.41)	0.242	0.269	0.069

Abbreviations: TC, Total cholesterol; TG, Triglyceride; LDL-C, Low density lipoprotein; HDL-C, High density lipoprotein; FGB, Fasting blood glucose; Hcy, Homocysteine; BMI, Body mass index; WC, Waist circumference; HC, Hip circumference; SBP, Systolic blood pressure; DBP, Diastolic blood pressure; HR, Heart rate.

Among the four measured anthropometric measurements, including body mass index (BMI), waist circumference (WC), hip circumference (HC) and waist/hip ratio, the mixed linear model showed a significant interaction of group by time and main effect of group on WC, HC and the waist/hip ratio; three (WC, HC and the waist/hip ratio) were significantly lower in the Baduanjin training group than in the control group at post-intervention or after the additional 12-week follow-up (Table 5).

For the systolic blood pressure (SBP) and diastolic blood pressure (DBP), the analysis of mixed linear model method showed obviously significant interaction effect of group by time (*P* < 0.001 and *P* = 0.023, respectively). The SBP and DBP values were significantly lower in the Baduanjin training group than in the control group at post-intervention (*P* < 0.001 for SBP and *P* = 0.044 for DBP), and this effect in SBP was maintained after the additional 12-week follow-up (*P* < 0.001). No significant main effect of group and interaction effect of group by time in heart rate was observed. These results are listed in Table 5.

### The Related Psychological outcomes

The measured psychological outcomes include mood, self-confidence, self-esteem, quality of life and sleep quality. A significant interaction effect of group by time and the main effect of group in all of those measured psychological outcomes were observed based on the mixed linear model analysis. In addition, we also found that all those outcomes in the Baduanjin training group were significantly superior to those in the controls at both post-intervention or after the additional 12-week follow-up (Table 6).

**Table 6:** The related psychological outcomes at all time points.

Variables	Time points	Control		Baduanjin		Mean difference (95% CI)	*P* value	Mixed linear model	
		n	Mean ± SE	n	Mean ± SE			*P*-value (between-groups)	*P*-value (Time × Group)
Mood (POMS)	Baseline	85	100.2 ± 1.61	85	103.2 ± 1.70	−2.98 (−7.61~1.66)	0.206		
	12-weeks	85	105.8 ± 1.63	85	92.61 ± 1.10	13.22 (9.35~17.10)	<0.001		
	24-weeks	85	104.3 ± 1.32	85	92.82 ± 1.14	11.45 (8.01~14.88)	<0.001	<0.001	<0.001
Self-confidence	Baseline	85	28.48 ± 0.32	85	27.92 ± 0.26	0.57 (−0.25~1.38)	0.173		
	12-weeks	85	27.69 ± 0.31	85	29.22 ± 0.25	−1.53(−2.31~−0.75)	<0.001		
	24-weeks	85	27.63 ± 0.27	85	28.76 ± 0.22	−1.13 (−1.81~−0.45)	0.001	0.037	<0.001
Self-esteem	Baseline	85	28.38 ± 0.26	85	28.08 ± 0.27	0.30 (−0.44~1.03)	0.430		
	12-weeks	85	27.79 ± 0.25	85	29.45 ± 0.24	−1.66 (−2.34~−0.98)	<0.001		
	24-weeks	85	27.66 ± 0.24	85	29.16 ± 0.23	−1.51 (−2.15~−0.86)	<0.001	0.002	<0.001
**Quality of life** (**SF-36)**									
SF-36 (Physical health)	Baseline	85	67.63 ± 1.73	85	64.18 ± 1.76	3.45 (−1.43~8.33)	0.165		
	12-weeks	85	59.52 ± 1.92	85	78.63 ± 1.26	−19.1 (−23.6~−14.7)	<0.001		
	24-weeks	85	59.26 ± 1.89	85	79.13 ± 1.21	−19.9 (−24.3~−15.4)	<0.001	<0.001	<0.001
SF-36 (Mind health)	Baseline	85	75.77 ± 1.55	85	72.46 ± 1.84	3.31 (−1.44~8.06)	0.171		
	12-weeks	85	71.11 ± 1.73	85	82.78 ± 1.16	−11.7 (−15.8~−7.55)	<0.001		
	24-weeks	85	70.41 ± 1.66	85	84.17 ± 1.07	−13.8 (−17.7~−9.87)	<0.001	<0.001	<0.001
**Sleep quality** (**Pitsburgh sleep quality index, PSQI)**									
PSQI	Baseline	85	7.68 ± 0.37	85	7.81 ± 0.38	−0.13 (−1.17~0.91)	0.806		
	12-weeks	85	7.99 ± 0.36	85	5.50 ± 0.25	2.49 (1.63~3.35)	<0.001		
	24-weeks	85	8.50 ± 0.38	85	5.85 ± 0.27	2.65 (1.74~3.57)	<0.001	<0.001	<0.001

### Adverse Events

No adverse events were reported during the exercise training or follow-up period.

## Discussion

### Main Findings

Compared with the control group, the 12-week Baduanjin training group showed a significant reduction in most of the cerebral haemodynamic parameters, particularly PI and RI, in nine measured cerebral arteries. Furthermore, most of those significant effects were sustained in the additional 12-week follow-up. We also found an obvious reduction in SBP, DBP, heart rate and anthropometric measurements, including WC, HC and the waist/hip ratio, and an obvious improvement in the related psychological or mental outcomes, including mood, self-confidence, self-esteem, sleep quality and quality of life, in the Baduanjin training group after the 12-week intervention or the additional 12-week follow-up.

### Cerebrovascular Haemodynamic Changes

Generally, normal ageing will cause marked structural and functional alterations in the cardiovascular and cerebrovascular systems, which may be linked to reductions in global CBF^37^. However, regular aerobic exercise can increase long-term CBF. The mechanisms are related to that aerobic exercise can enhance systemic arterial endothelial function, reduce large elastic artery stiffness and risk of arterial atherosclerosis in middle-aged and older adults^38,39^. The findings in this study show that the blood velocity of most measured cerebral arteries in the Badjanjin training group were significantly lower than in the control group. Those results seem to indicate that CBF is lower in the intervention group than in the control group, which conflicts with previous studies that showed that regular aerobic exercise was associated with elevated CBF in adults^40,41^. However, the resting CBF changes are tightly coupled to neuronal activity via adjustments in vascular resistance^15^, and they are easily influenced by physiological or psychological factors^42^. In our study, we also observed that the PI and RI of almost all measured arteries were significantly lower in the Baduanjin training group than in the control group. Furthermore, the observed psychological or mental variables including mood, self-confidence, self-esteem, sleep quality and quality of life were better in the Baduanjin training group than in the control group and may be a reasonable explanation for the findings in this study. In addition, low-intensity aerobic exercise may not directly affect the cerebrovascular blood velocity or cause CBF changes, and an increase in exercise intensity up to nearly 60% of the maximal oxygen uptake can result in elevations in CBF^43^. Ainslie PN, *et al*. reported that regular aerobic-endurance exercise (vigorous road running or cycling races more than 4 times per week) is associated with higher blood flow velocity in the MCA^40^. This study used rigorously screened healthy humans to avoid the known effects of prevalent age-related diseases on CBF. Another cross-sectional study also reported that over 150 minutes of moderate- to vigorous-intensity aerobic activity per week sustained during the adult lifespan can maintain aerobic fitness throughout the lifespan and improve cerebral haemodynamics later in life^44^. Baduanjin is a traditional Chinese Qigong exercise with a low exercise intensity. It consists of eight separate, delicate and smooth movements. Practising Baduanjin requires body-mind coordination by combining body posture, movement and breathing with meditation^45^. Therefore, we speculated that 12 weeks of regular Baduanjin training might improve cerebrovascular compliance by reducing the resistance of cerebral arteries due to relaxed body-mind training, thus resulting in a decrease in cerebral blood velocity.

### Cardiopulmonary Structure and Function

Cardiorespiratory capacity is typically associated with cerebrovascular diseases, and low cardiorespiratory capacity is an independent risk factor of ischaemic stroke^46^. Regular physical activity, particularly aerobic exercise, can substantially improve cardiorespiratory capacity, not only among healthy older adults but also among patients with chronic diseases, by improving cardiac structure, cardiac function and lung function^47–49^. However, no significant differences were found in the outcomes of cardiopulmonary structure and function between groups in the present study. A possible explanation is that the 12-week Baduanjin exercise intervention period may not have been sufficiently long to identify significant differences in those parameters among older community-dwelling adults at risk for ischaemic stroke.

### Blood Pressure, Heart Rate, Plasma Risk Factors and Anthropometric measurements

We observed significantly lower SBP after the 12-week Baduanjin training intervention and the additional 12-week follow-up. We also observed lower DBP and heart rate after 12 weeks of the Baduanjin intervention in community elderly adults at a high risk of ischaemic stroke in the Baduanjin training group compared to the control group. DBP and heart rate did not differ between the groups after the additional 12-week follow-up. High blood pressure in elderly adults is associated with an increased risk of ischaemic stroke and thus must be controlled. Li *et al*.^50^ did not observe effects on blood pressure in healthy adults with normal pressure after 16 weeks of Baduanjin training in their RCT, but a recent systematic review of 8 RCTs reported the beneficial effect of Baduanjin training combined with antihypertensive drugs versus antihypertensive drug use alone among patients with hypertension^32^. In our study, 54.1% of the participants (46/85) in the Baduanjin exercise group had hypertension, and 50.6% (43/85) of the patients in the control group had hypertension. Therefore, our findings indicate the efficacy of Baduanjin training as a management method for hypertension.

Plasma risk factors of ischaemic stroke include TC, TG, LDL, HDL, FBG and Hcy and were measured in our study, but no significant differences were found between groups except for the TC level, which was lower in the Baduanjin exercise group than in the control group at post-intervention. These findings were in line with those from the Li *et al*. study on healthy adults^48^ but in conflict with our previous systematic review regarding healthy adults or patients with hyperlipidaemia^33^. Additional studies are necessary to ascertain whether Baduanjin training can modulate blood lipids in adults with a high risk of ischaemic stroke.

Anthropometric measurements, including WC, HC and ratio of the waist/HCs, but not BMI, were significantly decreased in the Baduanjin training group compared to the control group. Li *et al*. found that a 16-week Baduanjin training intervention significantly decreased BMI and skinfold thickness at the abdomen in healthy adults in their RCT, whereas no significant differences were observed between groups for waist and hip parameters^50^.

### The Psychological or Mental Effect

To our knowledge, several previous studies have evaluated the effect of the Baduanjin training intervention on psychological outcomes among healthy adults or patients and have reported the positive beneficial results^30,31^. Chen *et al*. also observed that the 12-week Baduanjin training intervention could significantly improve sleep quality among community-dwelling older adults aged 60 years or older^29^. The findings in the present study are in accordance with the results of those previous studies.

### Strengths and Limitations of the Study

This is the first RCT to provide empirical evidence regarding the effectiveness of traditional Chinese Qigong-Baduanjin exercise on cerebral haemodynamic parameters; cardiopulmonary structure and function; traditional risk factors of CVD; and the related psychological outcomes of ischaemic stroke among older community adults at risk of ischaemic stroke. Our sample is representative of the population at risk for ischaemic stroke because all of them were recruited from the older population in the general community. A standardized and supervised Baduanjin exercise training programme makes it easy to reproduce. Furthermore, we had thorough recordings of exercise adherence and physical activity levels in the two groups. Second, no specific exercise intervention was used in the control group, which means that the between-group differences were due to the Baduanjin exercise training.

The main limitation of our study was that nine cerebral arteries with forty-five haemodynamic parameters (five parameters per artery) were selected as the primary outcomes, which may thereby increase the risk of detecting differences between groups by chance and making type I errors. Furthermore, we indirectly assessed CBF and cerebral vascular function using the cerebral haemodynamic parameters. Although it is well established that the blood flow velocity of some cerebral arteries may be considered a reliable indicator of cerebral perfusion at rest^51^, the positive changes in those haemodynamic parameters may not directly lead to improvements in CBF and cerebral vascular function. Second, a double-blinded study design was not possible due to the difficulty in administration and operation. Therefore, participants in the Baduanjin training group might have had higher expectations of the intervention effect. This awareness of intervention assignment might have introduced some bias into the results. Third, the 12-week follow-up assessment may not have been long enough to observe long-term effects, such as clinical events related to ischaemic stroke. Additional long-term randomized controlled trials should be conducted to determine whether and to what extent improvements in CBF and cerebral vascular function after Baduanjin training affect disease activity and the clinical events of ischaemic stroke in older community adults. Finally, Due to the difference of percentage of fibrillation atrial between the dropped out participants and the participants finished the study, it may cause to some extent confounding effect to the main results.

## Conclusion

This study demonstrated that Baduanjin significantly contributed to modulating cerebral haemodynamic parameters, reducing blood pressure and improving anthropometric parameters, mood, sleep quality and quality of life. Baduanjin exercise may be a useful and feasible strategy to reduce the risk of ischaemic stroke in older community populations.

## Data Availability

The datasets used and/or analysed during the current study are available from the corresponding author upon request.

## References

[1] Writing group members (2010). Heart disease and stroke statistics—2010 update: a report from the American Heart Association. Circulation..

[2] Roth GA (2015). Demographic and epidemiologic drivers of global cardiovascular mortality. N Engl J Med..

[3] Feigin VL (2015). Update on the global burden of ischemic and hemorrhagic stroke in 1990–2013: the GBD 2013 Study. Neuroepidemiology..

[4] Lackland DT (2014). Factors influencing the decline in stroke mortality: a statement from the American Heart Association/American Stroke Association. Stroke..

[5] Allen CL, Bayraktutan U (2008). Risk factors for ischaemic stroke. Int J Stroke..

[6] The European Stroke Organisation (ESO) Executive Committee and the ESO Writing Committee (2008). Guidelines for management of ischaemic stroke and transient ischaemic attack 2008. Cerebrovasc Dis..

[7] O’Donnell MJ (2010). Risk factors for ischaemic and intracerebral haemorrhagic stroke in 22 countries (the INTERSTROKE study): a case-control study. Lancet..

[8] Hankey GJ (2011). INTERSTROKE Study and the EPITHET Trial. Stroke: fresh insights into causes, prevention, and treatment. Lancet Neurol..

[9] Di LS, Koch G, Diomedi M, Stanzione P, Sallustio F (2012). Stroke prevention: managing modifiable risk factors. Stroke Res Treat..

[10] Zafar A, Al-Khamis FA, Al-Bakr AI, Alsulaiman AA, Msmar AH (2016). Risk factors and subtypes of acute ischemic stroke. A study at King Fahd Hospital of the University. Neurosciences (Riyadh)..

[11] Gupta A (2012). Cerebrovascular reserve and stroke risk in patients with carotid stenosis or occlusion: a systematic review and meta-analysis. Stroke..

[12] Bramlett HM, Dietrich WD (2004). Pathophysiology of cerebral ischemia and brain trauma: similarities and differences. J Cereb Blood Flow Metab..

[13] Bor-Seng-Shu E (2012). Cerebral hemodynamics: concepts of clinical importance. Arq Neuropsiquiatr..

[14] Meng L (2015). Cardiac output and cerebral blood flow: the integrated regulation of brain perfusion in adult humans. Anesthesiology..

[15] Grüne F, Kazmaier S, Stolker RJ, Visser GH, Weyland A (2015). Carbon dioxide induced changes in cerebral blood flow and flow velocity: role of cerebrovascular resistance and effective cerebral perfusion pressure. J Cereb Blood Flow Metab..

[16] Paulson OB, Strandgaard S, Edvinsson L (1990). Cerebral autoregulation. Cerebrovasc Brain Metab Rev..

[17] Kim YS (2008). Dynamic cerebral autoregulatory capacity is affected early in Type 2 diabetes. Clin Sci (Lond)..

[18] Muller M (2012). Hypertension and longitudinal changes in cerebral blood flow: the SMART-MR study. Ann Neurol..

[19] Yu, H., Huang, G.P., Yang, Z., Liang, F. & Ludwig, B. The Influence of Normal and Early Vascular Aging on Hemodynamic Characteristics in Cardio- and Cerebrovascular Systems. *J Biomech Eng*. **138**, 10.1115/1.4033179 (2016). 10.1115/1.4033179 27019876

[20] Suri MF (2016). Prevalence of Intracranial Atherosclerotic Stenosis Using High-Resolution Magnetic Resonance Angiography in the General Population: The Atherosclerosis Risk in Communities Study. Stroke..

[21] Zhang S (2013). Prevalence and risk factors of asymptomatic intracranial arterial stenosis in a community-based population of Chinese adults. Eur J Neurol..

[22] Haskell WL (2007). Physical activity and public health: updated recommendation for adults from the American College of Sports Medicine and the American Heart Association. Circulation..

[23] Eckel RH (2014). 2013 AHA/ACC guideline on lifestyle management to reduce cardiovascular risk: a report of the American College of Cardiology/American Heart Association Task Force on Practice Guidelines. Circulation..

[24] Kernan WN (2014). Guidelines for the prevention of stroke in patients with stroke and transient ischemic attack: a guideline for healthcare professionals from the American Heart Association/American Stroke Association. Stroke..

[25] Koh TC (1982). Baduanjin—an ancient Chinese exercise. Am J Chin Med..

[26] Zou, L., Wang, C., Chen, X. & Wang, H. Baduanjin Exercise for Stroke Rehabilitation: A Systematic Review with Meta-Analysis of Randomized Controlled Trials. *Int J Environ Res Public Health*. **15**, 600 (2018). 10.3390/ijerph15040600 PMC5923642 29584623

[27] Zou L (2018). Mindfulness-Based Baduanjin Exercise for Depression and Anxiety in People with Physical or Mental Illnesses: A Systematic Review and Meta-Analysis. Int J Environ Res Public Health..

[28] Zeng YG, Zhou XQ, Wang AL, Yang B, Wang ST (2005). Research on the impacts of fitness Qigong Baduanjin on figure and physical function among the middle-aged and aged people. J Beijing Sport Univ..

[29] Chen MC, Liu HE, Huang HY, Chiou AF (2012). The effect of a simple traditional exercise programme (Baduanjin exercise) on sleep quality of older adults: a randomized controlled trial. Int J Nurs Stud..

[30] Cheng FK (2015). Effects of Baduanjin on mental health: a comprehensive review. J Bodyw Mov Ther..

[31] Wu YM, Lin KL, Chen RF (2008). Research the intervention with Baduanjin exercise and healthy education to the 175 plasma glucose of diabetes mellitus subhealthy state. Chin Primary Health Care..

[32] Xiong X, Wang P, Li S, Zhang Y, Li X (2015). Effect of Baduanjin exercise for hypertension: a systematic review and meta-analysis of randomized controlled trials. Maturitas..

[33] Mei L, Chen Q, Ge L, Zheng G, Chen J (2012). Systematic review of chinese traditional exercise baduanjin modulating the blood lipid metabolism. Evid Based Complement Alternat Med..

[34] Zheng, G. *et al*. Primary prevention for risk factors of ischemic stroke with Baduanjin exercise intervention in the community elder population: study protocol for a randomized controlled trial. *Trials*. **15**, 113, doi: 10.1186 (2014). 10.1186/1745-6215-15-113 PMC3996200 24712684

[35] Wang, L. D. Screening of subjects at high risk for stroke [M]. Beijing: Screening and Prevention Program of Stroke Organized by Ministry of Public Health Press. 2–4 (2012).

[36] Health Qigong Management Center of General Administration of Sport of China: Health Qigong-Baduanjin. Beijing: People’s Sports Publishing House of China (2003).

[37] Niehaus L, Lehmann R, Röricht S, Meyer BU (2001). Age-related reduction in visually evoked cerebral blood flow responses. Neurobiol Aging..

[38] Ainslie PN (2008). Elevation ctivity prevents age-related impai with aerobic fitness throughout healthy human ageing. J Physiol..

[39] Taddei S (2000). Physical activity prevents age-related impairment in nitric oxide availability in elderly athletes. Circulation..

[40] Ainslie PN (2008). Elevation in cerebral blood flow velocity with aerobic fitness throughout healthy human ageing. J Physiol..

[41] Wang Y, Li M, Dong F, Zhang J, Zhang F (2015). Physical exercise-induced protection on ischemic cardiovascular and cerebrovascular diseases. Int J Clin Exp Med..

[42] Nagata K (2016). Cerebral circulation in aging. Ageing research review..

[43] Querido JS, Sheel AW (2007). Regulation of cerebral blood flow during exercise. Sports Med..

[44] Bailey DM (2013). Elevated aerobic fitness sustained throughout the adult lifespan is associated with improved cerebral hemodynamics. Stroke..

[45] Zheng G (2013). The effect of Baduanjin exercise for physical and psychological wellbeing of college students: study protocol for a randomized controlled trial. Trials..

[46] Kodama S (2009). Cardiorespiratory fitness as a quantitative predictor of all-cause mortality and cardiovascular events in healthy men and women: a meta-analysis. JAMA..

[47] Mahdiabadi J, Gaeini AA, Kazemi T, Mahdiabadi MA (2013). The effect of aerobic continuous and interval training on left ventricular structure and function in male non-athletes. Biol Sport..

[48] Spruit MA (2016). COPD and exercise: does it make a difference?. Breathe (Sheff)..

[49] Platt C, Houstis N, Rosenzweig A (2015). Using exercise to measure and modify cardiac function. Cell Metab..

[50] Li R (2014). The effect of baduanjin on promoting the physical fitness and health of adults. Evid Based Complement Alternat Med..

[51] Serrador JM, Picot PA, Rutt BK, Shoemaker JK, Bondar RL (2000). MRI measures of middle cerebral artery diameter in conscious humans during simulated orthostasis. Stroke..

